# Collaborative cross strain CC011/UncJ as a novel mouse model of T2-high, severe asthma

**DOI:** 10.1186/s12931-023-02453-y

**Published:** 2023-06-09

**Authors:** Lauren J. Donoghue, Kathryn M. McFadden, Daniel Vargas, Gregory J. Smith, Robert M. Immormino, Timothy P. Moran, Samir N. P. Kelada

**Affiliations:** 1 Department of Genetics, University of North Carolina at Chapel Hill, Chapel Hill, NC USA; 2 Curriculum in Genetics and Molecular Biology, University of North Carolina at Chapel Hill, Chapel Hill, NC USA; 3 Department of Pediatrics, Division of Allergy and Immunology, School of Medicine, University of North Carolina, Chapel Hill, NC USA; 4 Center for Environmental Medicine, Asthma and Lung Biology, University of North Carolina at Chapel Hill, Chapel Hill, NC USA

## Abstract

Among asthmatics, there is significant heterogeneity in the clinical presentation and underlying pathophysiological mechanisms, leading to the recognition of multiple disease endotypes (e.g., T2-high vs. T2-low). This heterogeneity extends to severe asthmatics, who may struggle to control symptoms even with high-dose corticosteroid treatment and other therapies. However, there are limited mouse models available to model the spectrum of severe asthma endotypes. We sought to identify a new mouse model of severe asthma by first examining responses to chronic allergen exposure among strains from the Collaborative Cross (CC) mouse genetics reference population, which contains greater genetic diversity than other inbred strain panels previously used for models of asthma. Mice from five CC strains and the often-used classical inbred strain BALB/cJ were chronically exposed to house dust mite (HDM) allergen for five weeks followed by measurements of airway inflammation. CC strain CC011/UncJ (CC011) exhibited extreme responses to HDM including high levels of airway eosinophilia, elevated lung resistance, and extensive airway wall remodeling, and even fatalities among ~ 50% of mice prior to study completion. Compared to BALB/cJ mice, CC011 mice had stronger Th2-mediated airway responses demonstrated by significantly elevated total and HDM-specific IgE and increased Th2 cytokines during tests of antigen recall, but not enhanced ILC2 activation. Airway eosinophilia in CC011 mice was completely dependent upon CD4^+^ T-cells. Notably, we also found that airway eosinophilia in CC011 mice was resistant to dexamethasone steroid treatment. Thus, the CC011 strain provides a new mouse model of T2-high, severe asthma driven by natural genetic variation likely acting through CD4^+^ T-cells. Future studies aimed at determining the genetic basis of this phenotype will provide new insights into mechanisms underlying severe asthma.

**Supplementary Information:**

The online version contains supplementary material available at 10.1186/s12931-023-02453-y.

## Introduction

The clinical presentation of asthma is characterized by shortness of breath, airway hyperresponsiveness, and reversible airflow obstruction. However, the molecular and cellular processes that lead to these clinical endpoints vary among asthmatics, leading to the classification of multiple asthmatic “endotypes” [1, 2]. For example, individuals vary in their airway inflammatory profile (e.g., T2-high vs. T2-low) and response to corticosteroid or biologic therapies [3]. Individuals with steroid-resistant asthma represent a patient population with unmet therapeutic need, accounting for > 50% of health care utilization costs in the US despite comprising < 10% of asthmatics [4]. Although asthma risk is known to be driven by a combination of genetic and environmental factors, the risk factors for steroid-resistant asthma are not completely understood [5]. Thus, there remains significant need to elucidate the cellular and molecular drivers of steroid-resistant endotypes to inform the design of personalized therapeutics [1, 2].

Mouse models of asthma have provided numerous insights into the mechanisms of allergic airways disease including those that may influence steroid-resistance [6, 7]. The steroid-resistant phenotype that defines severe asthma can be recapitulated in mice using various models including exposing mice to multi-allergen cocktails (i.e. ovalbumin, house dust mite (HDM) or cockroach extract, *Alternaria*) [8] or allergens supplemented with novel adjuvants (e.g., c-di-GMP) [9], and through genetic manipulation of genes with roles in airway inflammation (e.g., *STAT6, IL4, IL5, IL13*) [10]. Many mouse models of severe asthma aim to elicit neutrophil-dominant or T2-low inflammation of the airways, matching the presentation of a large and replicable subset of patients with severe disease [3, 9]. However, 33% of severe asthmatics have persistent T2 inflammation, characterized by sputum eosinophilia, elevated fraction of exhaled nitric oxide, and increased serum periostin [11, 12], highlighting the need for a spectrum of severe asthma models [13].

Given the significant contribution of genetic variation to asthma [14, 15], we surveyed mouse strains from the Collaborative Cross (CC) genetic reference population for extreme responses to chronic house dust mite allergen (HDM) exposure to identify new mouse models of severe asthma. The CC is a panel of recombinant inbred strains whose founder strains vary by over 45 million single nucleotide polymorphisms [16] and have been used to identify novel mouse models of human disease [17–19]. As such, we surveyed multiple CC strains in a model of repeated HDM exposure to test whether any strain exhibited stronger allergic inflammation phenotypes than BALB/cJ mice, which are often used in models of allergy and are considered Th2 prone. Compared to BALB/cJ mice, mice from the CC011/UncJ strain (hereafter referred to as “CC011”), exhibited significantly stronger Th2-mediated airway inflammation, which was also found to be CD4^+^ T-cell dependent. More intriguingly, CC011 mice exhibited phenotypes of severe asthma characterized by fatalities, elevated lung resistance, extensive airway wall remodeling with the presence of mast cells in the sub-mucosa, and steroid-resistance.

## Materials and methods

### Mice, allergen exposure, and corticosteroid treatment

We obtained BALB/cJ mice from The Jackson Laboratory (Bar Harbor, ME) and Collaborative Cross (CC) mice from the Systems Genetics Core at the University of North Carolina (Chapel Hill, NC). Mice from the five CC strains used for the initial survey (CC003, CC004, CC011, CC068, CC071) were obtained in 2017 and were all female. Additional female and male CC011 mice were obtained between 2018 and 2020. All mice used were housed on ALPHA-Dri bedding. For the allergen exposure models, mice were 7–11 weeks of age at the start of the protocol. We used a five-week exposure model consisting of intranasal administration of either 25 μL phosphate buffered saline (PBS, Gibco) or 25 μg of house dust mite (HDM) extract (Lot number 322781, 5.8 endotoxin units per 25 μg dose of HDM, Stallergenes Greer, Lenoir, NC) resuspended in 25 μL of PBS for 3 days per week. For mediastinal lymph node experiments, mice were exposed to PBS of HDM in the same manner for 3 days per week for 2 weeks and lymph nodes were harvested 24 h following the final exposure. To test the effect of corticosteroid treatment on allergen-induced phenotypes, mice were exposed in an abbreviated model for three weeks where mice received 25 μg (in 25 μL) of HDM by intranasal administration 3 days per week and during the third week of exposures also received either PBS or 1 mg/kg of dexamethasone (Aspen Pharmacare, Durban, South Africa) by intraperitoneal injection (i.p.) in 200 μL at the time of allergen exposure. All experiments with mice were compliant with a protocol approved by the UNC Institution Animal Care and Use Committee and were conducted at a facility approved and accredited by the Association for Assessment and Accreditation of Laboratory Animal Care International.

### Bronchoalveolar lavage

In most experiments (except as noted), bronchoalveolar lavage (BAL) was performed seventy-two hours after the last HDM exposure. For the survey with BALB/cJ and five CC strains, the left lung lobe was ligated and lavage was performed on the right lobes by instilling 600 μL of PBS with protease inhibitors. For all other BAL collections, both the right and left lungs together were instilled with two fractions of 1 mL of PBS with protease inhibitors. BAL fluid was centrifuged at 1000 × *g* for 5 min to pellet cells. Cells were resuspended in 250 μL of Hanks Balanced Salt Solution (Gibco), 100 μL of the suspension was spun onto slides, and slides were stained with Kwik Diff stains (Thermo Scientific). Differential cell counts were counted by investigators blinded to strain and treatment.

### Airway physiology

Mice were anesthetized with urethane (2 mg/kg) and tracheostomized with an 18-gauge, beveled cannula. Mice were treated with 0.8 mg/kg pancuronuium bromide and connected to a Buxco FinePointe RC System (Data Sciences International). After 5 min of acclimation, mice were administered nebulized PBS followed by methacholine resuspended in PBS at doses of 6.25, 12.5 and 25 mg/mL. Measurements of lung resistance were recorded every 2 s for 3 min following dose nebulization. The average resistance values over each 3-min recording period were subsequently used for statistical analyses and plotting.

### Histology

Immediately following death due to allergen exposure, lungs from CC011 mice were excised and fixed in 10% neutral buffered formalin. For mice exposed to PBS for 5 weeks, lungs were excised 72 h post final exposure and fixed in 10% neutral buffered formalin. Lungs were cut in 2 mm cross sections along the main stem bronchus starting at the hilum. Sections were embedded in paraffin, cut in 5 micron sections, and stained with hematoxylin and eosin (H&E), Alcian-blue and Periodic acid-Schiff (AB-PAS), or Mason’s Trichrome. Immunohistochemistry was performed for α-smooth muscle actin (α-SMA, Abcam ab124964). Sections from the most proximal lung section were imaged on an Olympus BX60F5 microscope with CellSens Standard software (Olympus Life Sciences).

### Plasma antibodies

Total IgE and HDM-specific IgE from plasma were measured from blood collected from the descending aorta using BD OptEIA Set Mouse IgE ELISA kits (cat. 555248, Becton Dickinson). Measurements for total plasma IgE were made following the manufacturers protocol and referenced to a standard curve with purified mouse IgE provided in the kit. To measure HDM-specific IgE, plasma samples were run through Ab SpinTrap Columns (GE Healthcare Life Sciences) to deplete IgG prior to using the OptEIA ELISA kit with plates coated with 100 μg/mL HDM. Values are expressed as optical density given the lack of standard for HDM-specific IgE.

### Mediastinal lymph node culture and cytokine measurement

Following two weeks of exposure to either PBS or HDM, mice were euthanized and mediastinal LNs (mLNs) were harvested for ex vivo cytokine analysis. Cells harvested from mLNs were maintained on ice in RPMI-1H (RPMI-1640, 1% fetal bovine serum, penicillin–streptomycin, 50 μM 2-mercaptoethanol, 10 mM HEPES) to maintain viability, then passed through a 70 μm strainer to obtain single cell suspensions. 200,000 cells/well were cultured for 4 days in complete IMDM-10 medium (IMDM, 10% fetal bovine serum (Gemini, West Sacramento, CA), penicillin–streptomycin, 50 μM 2-mercaptoethanol) and 10 μg/mL of the HDM allergen. Cell culture supernatants were collected 4 days later for measurement of IL-4, IL-5, IL-13, IL-17A, and IFN-γ by ELISA using custom capture and detection antibody sets. Capture and detection antibodies for IL4, IL17A, and IFN-γ were purchased from BioLegend (San Diego, CA), antibodies for IL-13 were purchased from eBioscience (Thermo Fisher scientific, Waltham, MA), and antibodies for IL-5 were purchased from BD (Franklin Lakes, NJ). Cytokine standards were purchased from Peprotech (Cranbury, New Jersey) or Gemini (Sacramento, CA).

### Flow cytometry analysis of lung type 2 innate lymphoid cells (ILC2s)

BALB/cJ or CC011 mice were intranasally exposed to PBS or 25 µg HDM every 2–3 days for 3 total treatments. At 24 h following the last treatment, lung cells were isolated and analyzed by flow cytometry as previously described[20]. Briefly, harvested lungs were minced and digested with Liberase TM (100 μg/mL; Roche, Indianapolis, IN), collagenase XI (250 μg/ml), hyaluronidase 1a (1 mg/ml), and DNase I (200 μg/ml; Sigma) for 1 h at 37 °C. The digested tissue was passed through a 70 µm nylon strainer to obtain a single cell suspension. RBCs were lysed with 0.15 M ammonium chloride and 1 mM potassium bicarbonate. Lung cells were then cultured with PMA (50 ng/mL), ionomycin (1 ug/mL), and 1X BD GolgiPlug™ (BD Biosciences) for 3 h at 37 °C/5% CO_2_. For antibody staining of surface antigens, cells were incubated with anti-mouse CD16/CD32 (2.4G2) for 5 min to block Fc receptors, and then stained with biotinylated monoclonal antibodies (mAb) for lineage (Lin) markers [CD3ε (145-2C11), CD4 (GK1.5), CD8α (53–6.7), CD11b (M1/70), CD11c (N418), CD19 (6D5), CD49b (DX5), Gr-1 (RB6-8C5), TCRβ (H57-597), TCRγδ (GL3), TER-119], FITC anti-ICOS mAb (C398.4A), PE-Cy7 anti-ST2 mAb (DIH9), and Alexa Fluor® 700 anti-CD45 mAb for 30 min on ice. Anti-IL-13 mAb was purchased from Invitrogen (Waltham, MA); all other antibodies were purchased from BioLegend (San Diego, CA). Cells were subsequently stained with Brilliant Violet 421™ streptavidin (BioLegend) and the viability dye Zombie Aqua™ (BioLegend) for 20 min on ice. The cells were then fixed and permeabilized using the BD Fixation/Permeabilization Solution Kit (BD Biosciences) per the manufacturer’s instructions, and stained with PE anti-IL-13 mAb (eBio13A) and APC anti-IL-5 mAb (TRKF5) at room temperature for 30 min. Flow cytometry data was acquired with a 4 laser LSRII (BD Biosciences) and analyzed using FlowJo (Treestar, Ashland, OR) software. Only single cells were analyzed. Innate lymphoid cells were identified as Lin-CD45 + ICOS + ST2 + cells.

### ***Antibody-mediated depletion of CD4***^+^***T cells***

CC011 female and male mice received i.p. injections of 0.5 mg GK1.5 mAb (BioXCell, Lebanon, NH) or control rat IgG daily for three consecutive days and then twice weekly thereafter to maintain depletion of CD4^+^ cells [21]. Pilot studies confirmed that this treatment protocol resulted in ~ 99% depletion of CD4^+^ T cells in CC011 mice (data not shown). Starting six days after the initial antibody treatments, mice were exposed intranasally to PBS or 25 µg HDM three times weekly for two weeks as described above. BAL was collected 24 h after the final PBS or HDM treatment and cell differentials were determined as above.

### Data availability

Raw data used in each figure are provided in the accompanying Additional file 1: Table S1–S11.

## Results

### CC011 mice have extreme airway eosinophilia and fatalities in response to chronic allergen exposure

**Fig. 1:**
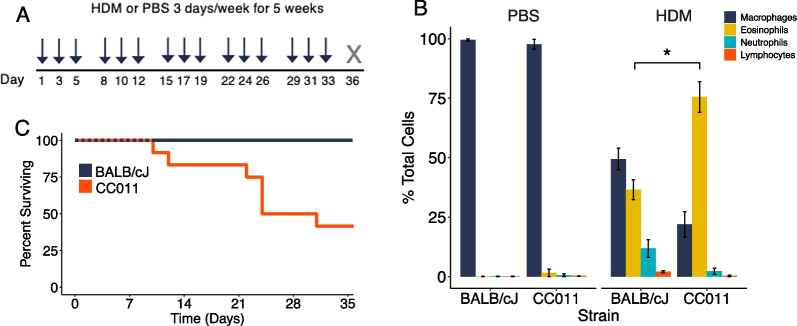
CC011 mice exhibit extreme eosinophilic airway inflammation and fatalities due to chronic allergen exposure. **A** Chronic intranasal allergen exposure model. HDM or PBS delivered to mice intranasally (3 days/week for 5 weeks (arrows)). Mice were sacrificed 72 h after final exposure (gray X). **B** BAL differential cell type percentages in PBS and HDM-exposed BALB/cJ and CC011 mice (n = 2/strain PBS, 3–4/strain HDM). Significance of cell-type matched *t*-tests between BALB/cJ and CC011 mice denoted as * for *P* < 0.01. **C** Survival curve comparing HDM-exposed BALB/cJ vs. CC011 mice (n = 20–23/strain)

 To identify a new mouse model of severe asthma, we first administered HDM or saline (PBS) intranasally to BALB/cJ mice and mice from five CC strains for three days per week for five weeks (Fig. 1A). Unsurprisingly, there were significant effects of strain on allergen-induced eosinophil, neutrophil, and lymphocyte counts in bronchoalveolar lavage (BAL) fluid (ANOVA *P* < 0.01, Additional file 2: Fig. S1). CC011 mice stood out with an extremely high level of eosinophilic inflammation, with eosinophils comprising ≥ 75% of cells in BAL, more than double that observed in BALB/cJ mice (Fig. 1B). More impressively, during these experiments, we found that some CC011 mice died immediately following allergen administration after only two or more weeks of exposure. This finding was reproducible across subsequent experiments including both female and male CC011 mice with 50% mortality on average prior to completing the five-week protocol (Fig. 1C). No PBS exposed CC011 mice died during these experiments, nor did they exhibit elevated eosinophil or neutrophil counts in BAL (Fig. 1B).

### Lung physiology is altered in control and allergen exposed CC011 mice

**Fig. 2:**
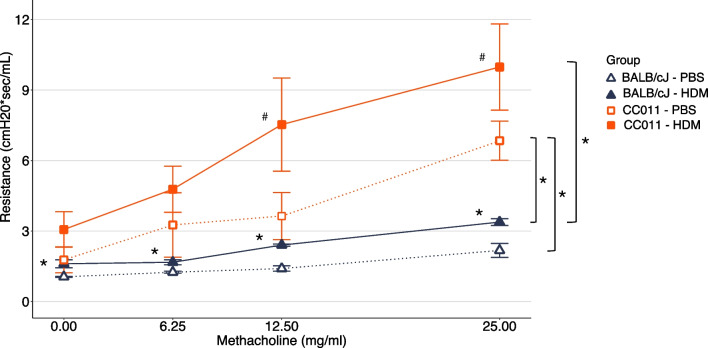
CC011 mice have greater total lung resistance under both HDM and PBS conditions compared to BALB/cJ. Total lung resistance to increasing doses of methacholine. n = 3–6 HDM exposed and n = 3–4 PBS-exposed mice per strain. Within strain treatment contrasts from Welch’s one-sided *t*-test denoted by * for *P* < 0.05 and ^#^ for *P* < 0.1. Additional statistically significant (*P* < 0.05) differences between PBS exposed CC011 mice and both BALB/cJ treatment groups at 25 mg/ml methacholine are shown on the right

Given the striking inflammation and mortality in CC011, we sought to determine if these extreme responses were associated with differences in lung physiology as compared to BALB/cJ mice. We measured lung resistance during methacholine challenge and found that HDM-exposed CC011 mice had greater total lung resistance that CC011 mice treated with PBS (*P* < 0.1) and significantly greater resistance compared to HDM-exposed BALB/cJ mice (*P* < 0.05, Fig. 2). Due to the ~ 50% mortality in CC011 mice during chronic allergen exposure, only 3 HDM-exposed mice survived the entire protocol before phenotyping, and therefore the resistance values reported in Fig. 2 likely underestimate the true response of CC011 mice. Additionally, total lung resistance was greater among PBS-exposed CC011 mice compared to BALB/cJ mice exposed to either PBS or HDM, indicating that even without allergen-induced inflammation, CC011 mice exhibit heightened airway reactivity.

### CC011 mice exhibit severe airway wall remodeling and pathology following chronic allergen exposure

**Fig. 3:**
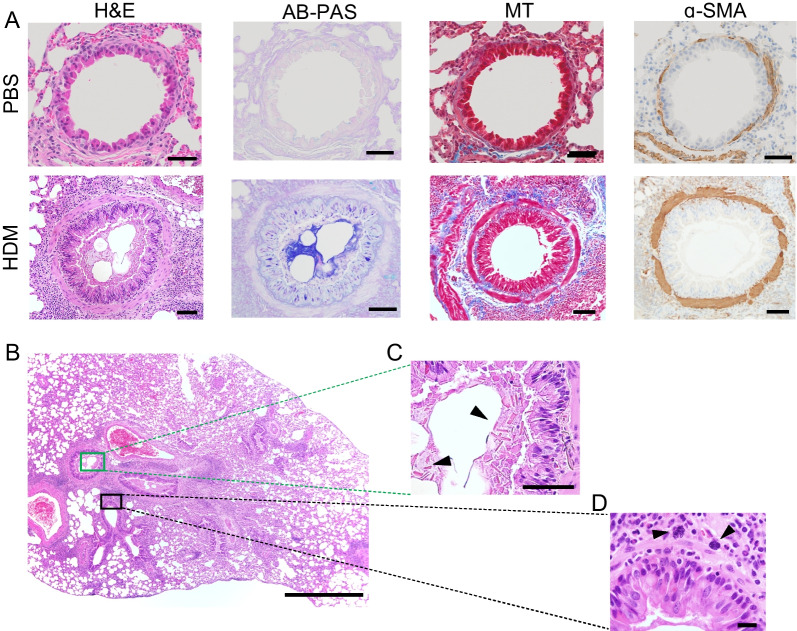
CC011 mice chronically exposed to HDM exhibit histological features of severe asthma. **A** Representative cross-sections of large airways from CC011 control mice exposed to PBS for 5 weeks (top row) or collected immediately upon death from mice that succumbed to repeated HDM-exposure (bottom row). Large airway stained (from left to right) with H&E, Alcian Blue-Periodic Acid-Schiff (AB-PAS), Mason’s Trichrome (MT), and α-SMA by immunohistochemistry. Scale bars = 50 μM. **B**–**D** Additional abnormalities in HDM mouse shown in A with H&E staining. **B** H&E of whole lung cross-section. Scale bar = 500 μM. **C** Higher magnification image of airway with arrowheads pointing to extracellular crystals. Scale bars = 50 μM. **D** Higher magnification image with arrowheads indicating submucosal mast cells. Scale bar = 10 μM

We examined airway remodeling in CC011 mice that died immediately after HDM treatment and found evidence of extensive airway wall remodeling, including mucous cell metaplasia, subepithelial fibrosis, and smooth muscle hypertrophy (Fig. 3A). Some airways were partially occluded by mucus and contained extracellular crystals similar to those reported in human asthma (Fig. 3C) [22]. Concordant with the BAL data, we also saw abundant peribronchiolar granulocytic inflammation (Fig. 3B). Additionally, we noted the presence of mast cells in the submucosa (Fig. 3D). Perivascular inflammation and vascular remodeling was also observed in these mice (Additional file 2: Fig. S2). None of these histological features were observed in PBS-exposed CC011 mice (Fig. 3A).

### CC011 mice have augmented T helper 2, but not ILC2, responses to HDM allergen

**Fig. 4:**
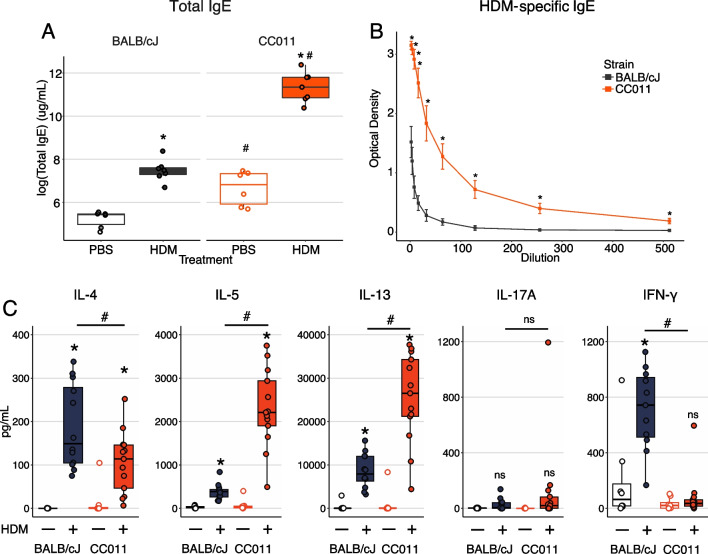
CC011 mice exhibit a stronger Th2-bias to HDM allergen than BALB/cJ mice. **A** Plasma total IgE from BALB/cJ and CC011 mice exposed to PBS or HDM for 5 weeks. n = 2–4/strain for PBS group (white-filled), n = 3–7/strain for HDM group (color-filled). Significance of *t*-tests for treatment effects within strain denoted by * for *P* < 0.05; significance of treatment-matched strain effects denoted as # for *P* < 0.05. **B** HDM-specific plasma IgE in BALB/cJ and CC011 mice exposed to HDM for 5 weeks. n = 3–7/strain. Significance of *t*-tests for treatment effects within strain denoted by * for *P* < 0.05. **C** Cytokine secretion following PBS or HDM stimulation in cultured mediastinal lymph nodes from BALB/cJ and CC011 mice exposed to HDM in 2-week model. n = 8/strain for PBS stimulated (white-filled) and n = 12–13/strain for HDM (color-filled) stimulated cultures. Significance of *t*-tests for treatment effects within strain denoted by * for *P* < 0.05; significance of *t*-tests for strain effects between HDM-exposed BALB/cJ and CC011 mice denoted by ^#^ for *P* < 0.05

We then compared features of Th2 immune responses between CC011 and BALB/cJ mice by examining plasma IgE in CC011 vs. BALB/cJ mice. Compared to BALB/cJ mice, CC011 mice had markedly elevated levels of total IgE in both PBS and HDM conditions as well as significantly higher HDM-specific IgE (Fig. 4A, B). We next compared HDM-specific T helper responses in lung-draining mediastinal lymph nodes (mLN) from CC011 and BALB/cJ mice following HDM exposure. Upon ex vivo stimulation with HDM allergen, mLN cells from HDM-exposed CC011 mice produced significantly higher levels of IL-5 and IL-13 compared to mLN from HDM-exposed BALB/c mice (Fig. 4C). Additionally, HDM-induced IFN-γ production was decreased in CC011 mice compared to BALB/c mice, consistent with enhanced Th2 skewing in CC011 mice, whereas HDM-induced IL-4 and IL-17A production was similar between CC011 and BALB/cJ mice (Fig. 4C).

**Fig. 5:**
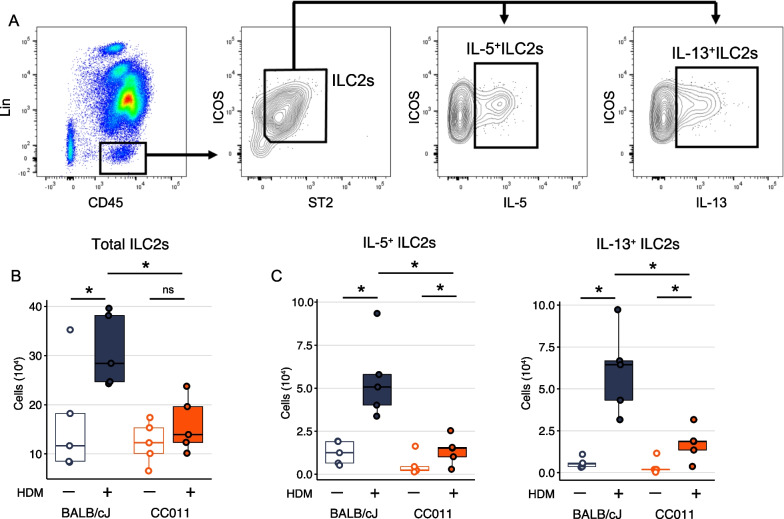
CC011 mice do not exhibit enhanced ILC2 responses to HDM compared to BALB/cJ mice. **A** Gating strategy for flow cytometric identification of ILC2s (Lin^−^CD45^+^ICOS^+^ST2^+^) from lung single cell suspensions. **B**, **C** Total number of lung ILC2s (**B**) from BALB/cJ and CC011 mice following three treatments with PBS or HDM (n = 5 mice per treatment). Significance of *t*-tests for treatment effects within strain or between HDM-exposed BALB/cJ and CC011 denoted by * for *P* < 0.05. **C** Number of IL-5^+^ and IL-13^+^ ILC2s as determined by intracellular cytokine staining following stimulation of cells with PMA and ionomycin

Group 2 innate lymphoid cells (ILC2) are important for the initiation of type 2 immune responses to inhaled allergens including HDM [23, 24]. Upon activation by epithelial-derived cytokines and alarmins, ILC2s produce IL-5 and IL-13, which contribute to eosinophilic airway inflammation [24]. ILC2s also help initiate allergen-specific Th2 responses by inducing migration of lung dendritic cells to draining lymph nodes through an IL-13-dependent mechanism [25]. Therefore we investigated if ILC2 responses were increased in CC011 mice compared to BALB/cJ mice following HDM exposure (Fig. 5A). Treatment of BALB/cJ mice with HDM resulted in increased numbers of lung ILC2s compared to PBS-treated mice (Fig. 5B). In contrast, treatment of CC011 mice with HDM did not significantly increase total numbers of ILC2s compared to PBS-treated mice (Fig. 5B). HDM treatment of BALB/cJ and CC011 mice increased the numbers of IL-5- and IL-13-producing ILC2s compared to PBS-treated mice (Fig. 5C), indicating the HDM exposure induced activation of lung ILC2s in both strains of mice. However, the numbers of total ILC2s and IL-5^+^ or IL-13^+^ lung ILC2s were significantly lower in HDM-exposed CC011 mice compared to HDM-exposed BALB/cJ mice (Fig. 5C). Moreover, the median fluorescence intensities (MFI) of IL-5 and IL-13 protein levels in ILC2s were similar between BALB/cJ and CC011 mice, indicating equivalent cytokine production at the cellular level (Additional file 2: Fig. S3). Taken together, these results suggest that the exaggerated T2 immune responses in CC011 mice are not explained by enhanced ILC2 activation.

### ***CD4***^+^***T cells are essential for HDM-induced allergic airway inflammation in CC011 mice***

**Fig. 6:**
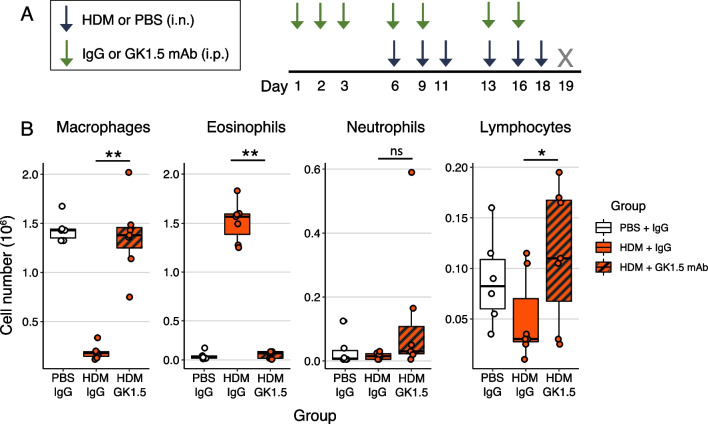
CD4^+^ T cell depletion completely abrogates eosinophilia in CC011 mice. **A** Schematic of CD4.^+^ T cell depletion treatment model for data shown in part B. **B** BAL differential counts from CC011 mice (n = 6–7/group) treated with PBS or HDM and IgG or GK1.5 mAb. Significance of ANOVA test for group effects followed by Tukey HSD test denoted by * for *P* < 0.05 or ** for *P* < 0.001

Given our findings that allergen-specific Th2 responses are enhanced in CC011 mice, we next investigated if CD4^+^ T helper cells are required for HDM-induced eosinophilic airway inflammation in CC011 mice. CC011 were treated with either anti-CD4 (GK1.5) mAb to deplete CD4^+^ T cells or control rat IgG antibody, and then exposed intranasally to HDM for 2 weeks to induce allergic airway inflammation (Fig. 6A). CD4-depletion completely abrogated HDM-induced eosinophilic airway inflammation in CC011 mice, as indicated by an absence of BAL eosinophils in mice treated with GK1.5 mAb compared to control rat IgG antibody (Fig. 6B). Thus, CD4^+^ T helper cells are necessary for HDM-induced allergic airway inflammation in CC011 mice.

### Allergic inflammation in CC011 mice is resistant to dexamethasone treatment

**Fig. 7:**
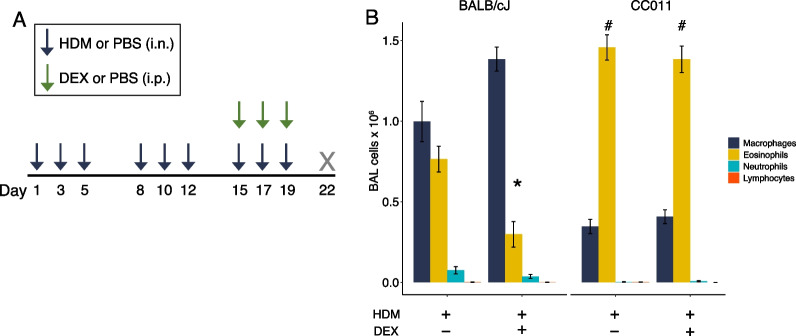
Extreme airway eosinophilia in CC011 mice is steroid-insensitive. **A** Exposure model with 3-week intranasal HDM allergen and dexamethasone (DEX) treatment. **B** BAL cells in BALB/cJ and CC011 exposed to HDM ± 1 mg/kg DEX treatment. * Denotes statistically significant contrast (*P* < 0.05) of within-strain DEX effect on eosinophil number. # Denotes statistically significant contrast (*P* < 0.05) of eosinophil numbers between strains of same treatment groups

Finally, we sought to determine if the severe, Th2 inflammatory responses in CC011 mice could be reduced with systemic treatment with the corticosteroid dexamethasone (DEX) in a shorter, three-week exposure model to limit the effects on mortality (Fig. 7A). In BALB/cJ mice exposed to HDM, DEX treatment significantly reduced eosinophilic inflammation by 60% (Fig. 7B). Strikingly, there was no reduction in eosinophilic inflammation in HDM-exposed CC011 mice treated with DEX (Fig. 7B).

## Discussion

To our knowledge, this is the first report of a mouse model of T2-high, steroid-resistant asthma in which fatalities occur reproducibly. Given that no single mouse model can capture the full range of clinical and molecular phenotypes of asthma [7], this model can help fill a critical need for severe asthma models driven by T2-high inflammation. Additionally, in comparison to other eosinophilic severe asthma mouse models, the susceptibility in CC011 mice is driven by natural genetic variation as opposed to a specialized exposure protocol or targeted genetic manipulations. We suspect the extreme phenotypes in CC011 are due to the additive or epistatic effects of multiple genes, as the extensive phenotypic variation observed across CC strains is largely attributable to the novel allele combinations uniquely present in each strain [26]. As such, the genetic architecture of CC011 mice may be more comparable to the polygenic origins of human asthma [5, 7]. Of note, CC011 has been previously reported to also have a spontaneous colitis disease phenotype [19], which could imply a generalized imbalance of immune-mucosal homeostasis in this strain. However, the colitis phenotype does not appear until > 20 weeks of age (while we observed death due to allergen exposure in mice as young as 9 weeks of age), and thus we anticipate the genetic architectures between these traits differ.

The airway wall remodeling features and submucosal mast cells observed in CC011 mice also mimic what has been observed in severe asthmatics, including those with T2-high disease [6, 27, 28]. It is plausible that the fatalities and high airway resistance to methacholine observed in CC011 are driven by the combinatorial effects of granulocytes as well as remodeling including the notable airway occlusion due to mucus [29]. The consequences of the observed vascular remodeling are less understood; however, thickening and increased muscularity of the vascular walls have been observed in fatal or severe asthma, with possible links to pulmonary hypertension [30, 31]. The elevated lung resistance in PBS-exposed CC011 mice that lacked granulocytic inflammation, however, indicates that this strain may have reduced airway caliber and/or abnormally reactive airway smooth muscle contractility, thus predisposing it to partial or complete airway closure after HDM-induced inflammation.

We observed that CC011 mice have significantly augmented HDM-specific Th2 responses relative to BALB/cJ mice, which are generally considered to be a Th2-biased inbred mouse strain when evaluated in asthma models [32]. Furthermore, antibody-mediated depletion studies demonstrated that HDM-induced airway eosinophilia was completely dependent on CD4^+^ T cells, suggesting the Th2 cells are key drivers of allergic airway inflammation in CC011 mice. The significantly increased production of IL-5 and IL-13, but not IL-4, by HDM-specific T helper cells suggests that pathogenic Th2 responses may be specifically enhanced in CC011 mice. Pathogenic Th2 cells, which express the IL-33 receptor and produce high levels of IL-5, are considered key players in the pathogenesis of allergic inflammation in both mice and humans [33]. The differential expression of IL-4, IL-5, and IL-13 by HDM-specific T helper cells from CC011 mice may also be due to strain-specific differences in transcriptional regulation of these cytokines [33]. For example, the transcription factor GATA3, which is necessary for Th2 cell differentiation, is essential for driving the expression of IL-5 and IL-13, but not IL-4, in established Th2 cells [34, 35]. Furthermore, the T-box transcription factor *Eomes* regulates the expression of IL-5, but not IL-4, in memory Th2 cells by inhibiting GATA3 binding to the *Il5* promoter region [36]. Thus, the differential activity of the aforementioned transcription factors may have a role. Future quantitative trait loci mapping studies will help determine if strain-specific differences in these transcription factors or others are associated with enhanced Th2 responses in CC011 mice. Innate cytokine signaling in Th2 cells can also differentially regulate the production of Th2 cytokines. For example, exposure of pathogenic Th2 cells to IL-33 can induce the production of IL-5, but not IL-4, through an ST2-dependent mechanism [37]. Whether IL-33-ST2 signaling is enhanced in pathogenic Th2 cells from CC011 is currently unknown and will be the focus of future studies.

ILC2s, which produce IL-5 and IL-13 during the early stages of type 2 immune responses, also play an important role in the initiation of Th2 responses and eosinophilic airway inflammation [25]. We did not observe increased numbers of total or activated ILC2s in CC011 mice compared to BALB/cJ mice following HDM exposure, suggesting that exaggerated ILC2 responses are not solely responsible for the enhanced Th2 priming observed in CC011 mice. However, assessment of epithelial cell-mediated innate immune responses and further phenotypic characterization of lung ILC2s will be necessary to determine if dysregulated innate immunity contributes to amplified Th2 responses in CC011 mice.

Determining the mechanisms underlying of the steroid-resistance phenotype in this model will also provide the opportunity to further understand the underpinnings of severe asthma. The mechanisms of steroid resistance in asthma are heterogenous and include direct actions of the glucocorticoid receptors (GR), such as decreased nuclear translocation of the GR-alpha isoform and increased expression of the antagonistic GR-beta isoform [2, 38]. It is possible that either or both of these features of GR-mediated signaling in CC011 mice is dysfunctional and not able to suppress T2-inflammation by endogenous or exogenous ligands. While T2-low airway inflammation is frequently associated with steroid-resistant asthma, severe asthma patients with T2-high airway inflammation can also exhibit steroid insensitivity in which ILC2s have been implicated as mediators [12, 39]. Airway ILC2s from asthmatic patients with increased thymic stromal lymphopoietin levels are resistant to dexamethasone [40]. However, we did not observe increased numbers of activated ILC2s in steroid-resistant CC011 mice compared to steroid-sensitive BALB/cJ mice, suggesting that dysregulation of ILC2s is not responsible for steroid-resistant airway inflammation in this model. Memory-type pathogenic CD4^+^ Th2 cells may also contribute to steroid-resistance in T2-high asthma. In a mouse model of IL-33-mediated eosinophilic airway inflammation, dexamethasone treatment failed to inhibit the number and activation of memory-type pathogenic Th2 cells [41]. Given that pathogenic Th2 responses appear to be augmented in CC011 mice, it is possible that these cells are the primary mediators of steroid resistance in these mice. Future studies interrogating the molecular and cellular mechanisms of steroid resistance in CC011 are clearly needed and may reveal novel therapeutic targets for severe T2-high asthma.

In conclusion, in the context of repeated HDM exposure, the CC011 strain provides a novel mouse model of severe T2-high asthma. Further interrogation of the genetic and immunologic factors leading to the extreme phenotypes observed in CC011 mice holds the potential to elucidate new insights into airway function and regulation of steroid-resistant, T2-inflammation.

## Supplementary Information

**Additional file 1: Tables S1-S11**. which contain the raw data generated and analyzed in this study.

**Additional file 2: Figures S1-S3. Figure S1. A **BAL differential cell type percentages in HDM-exposed BALB/cJ and CC mice. Significance of *t*-test between eosinophil percentage between BALB/cJ and CC strains denoted as * for *P* < 0.01. **B** BAL differential cell counts for data shown in main text Figure 1B. Significance of *t*-tests of  total cell counts and eosinophils between HDM-exposed BALB/cJ and CC011 mice are denoted by ^#^ for *P* < 0.1. Note BAL was performed on right lung lobes only. **Figure S2.** Perivascular inflammation and vascular remodeling in CC011 mice chronically exposed to HDM. Representative cross-sections of H&E stained lung tissue from CC011 mice exposed to PBS or HDM for 5 weeks. Tissue from HDM-exposed mice was collected immediately upon death due to repeated allergen exposure. Scale bars = 100uM. **Figure S3. **Median fluorescence intensity of IL-5 and IL-13 in ILC2s from BALB/cJ or CC011 mice, as determined by flow cytometry, following three treatments with PBS or HDM. Significance of *t*-tests for treatment effects within strain  are denoted by * for *P *< 0.05.

## Data Availability

All data reported this study are included in this published article and the supplementary files.

## Abbreviations

BAL: Bronchoalveolar lavage
CC: Collaborative Cross
DEX: Dexamethasone
HDM: House dust mite allergen
ILC2s: Group 2 innate lymphoid cells
mLN: Mediastinal lymph nodes
PBS: Phosphate buffered saline
Th2: T helper 2
