# Supplementary material for: Collaborative cross strain CC011/UncJ as a novel mouse model of T2-high, severe asthma
Source: Respir Res. 2023 Jun 9;24:153. doi: 10.1186/s12931-023-02453-y (PMC10251525; doi:10.1186/s12931-023-02453-y)
Supplement: Supplementary file 2 — Additional file 2: Figures S1-S3. Figure S1. A BAL differential cell type percentages in HDM-exposed BALB/cJ and CC mice. Significance of t-test between eosinophil percentage between BALB/cJ and CC strains denoted as * for P < 0.01. B BAL differential cell counts for data shown in main text Figure 1B. Significance of t-tests of total cell counts and eosinophils between HDM-exposed BALB/cJ and CC011 mice are denoted by # for P < 0.1. Note BAL was performed on right lung lobes only. Figure S2. Perivascular inflammation and vascular remodeling in CC011 mice chronically exposed to HDM. Representative cross-sections of H&E stained lung tissue from CC011 mice exposed to PBS or HDM for 5 weeks. Tissue from HDM-exposed mice was collected immediately upon death due to repeated allergen exposure. Scale bars = 100uM. Figure S3. Median fluorescence intensity of IL-5 and IL-13 in ILC2s from BALB/cJ or CC011 mice, as determined by flow cytometry, following three treatments with PBS or HDM. Significance of t-tests for treatment effects within strain are denoted by * for P < 0.05. [file 12931_2023_2453_MOESM2_ESM.pdf]

Additional file Figure S1

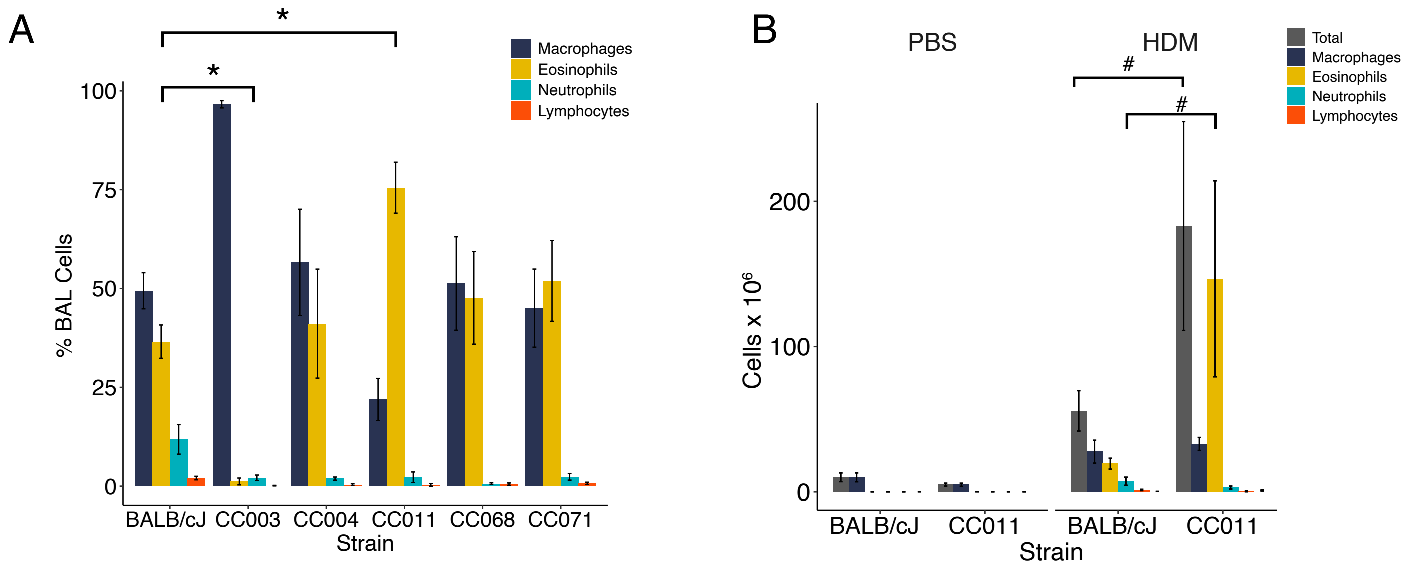

Figure S1. (A) BAL differential cell type percentages in HDM-exposed BALB/cJ and CC mice (n=3-4/strain). Significance of *t*-test between eosinophil percentage between BALB/cJ and CC strains denoted as * for $P < 0.01$. **(B)** BAL differential cell counts for data shown in main text Figure 1B. Significance of *t*-tests of total cell counts and eosinophils between HDM-exposed BALB/cJ versus CC011 mice are denoted by $^{\#}$ for $P < 0.1$. Note BAL was performed on right lung lobes only.

Additional file Figure S2

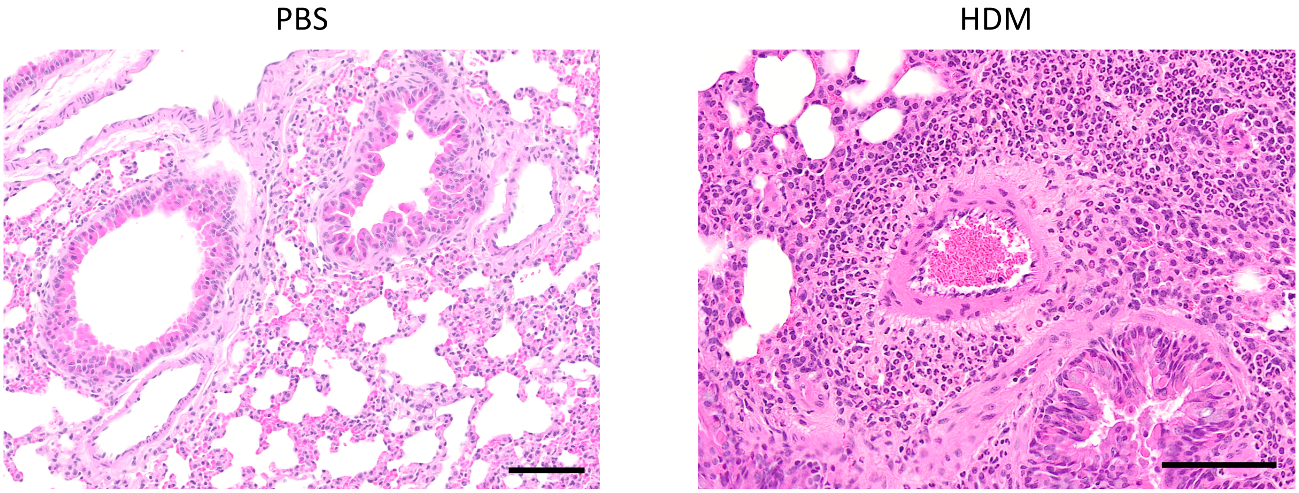

Figure S2. Perivascular inflammation and vascular remodeling in CC011 mice chronically exposed to HDM. Representative cross-sections of H&E stained lung tissue from CC011 mice exposed to PBS (left) or HDM (right) for 5 weeks. Tissue from HDM-exposed mice was collected immediately upon death due to repeated allergen exposure. Scale bars = 100uM.

Additional file Figure S3

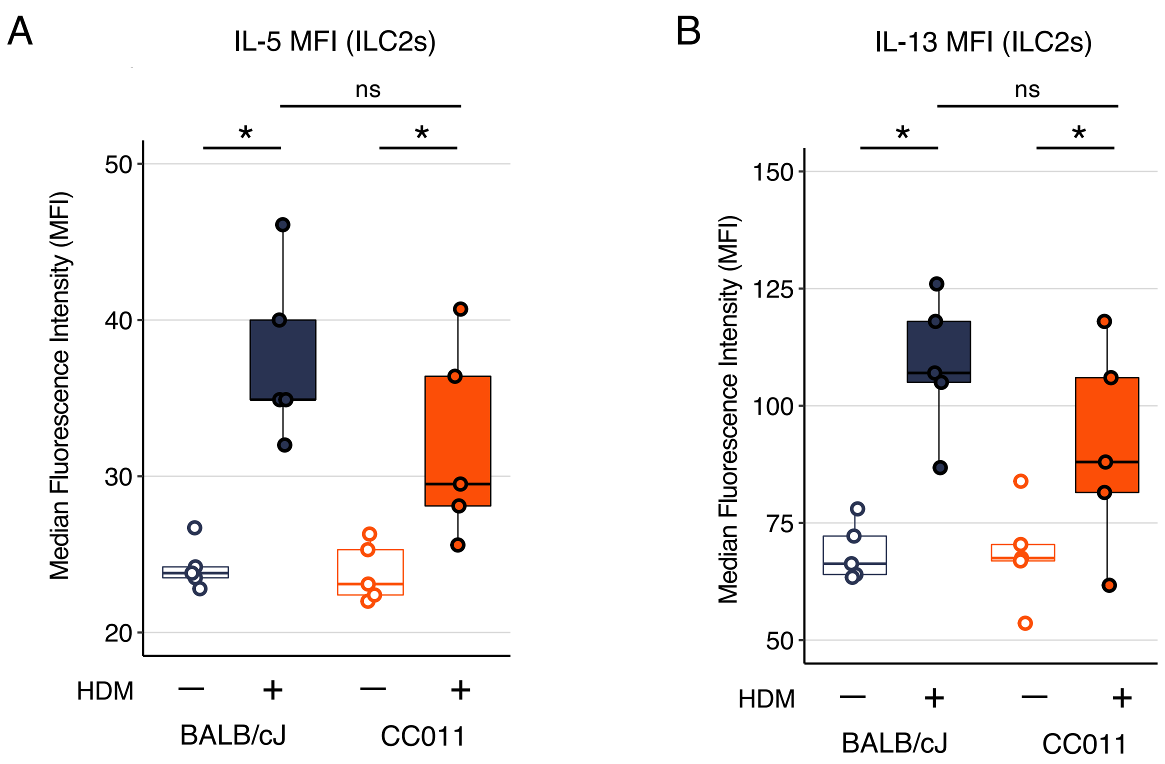

Figure S3. Median fluorescence intensity of **(A)** IL-5 and **(B)** IL-13 in ILC2s from BALB/cJ or CC011 mice, as determined by flow cytometry, following three treatments with PBS or HDM (n=5 mice per treatment). Significance of *t*-tests for treatment effects within strain denoted by * for $P < 0.05$. ns: not significant.
